# Serum Amyloid A (SAA) and Its Interaction with High-Density Lipoprotein Cholesterol (HDL-C): A Comprehensive Review

**DOI:** 10.3390/ijms27010241

**Published:** 2025-12-25

**Authors:** Angela P. Moissl-Blanke, Graciela E. Delgado, Bernhard K. Krämer, Rüdiger Siekmeier, Daniel Duerschmied, Winfried März, Marcus E. Kleber

**Affiliations:** 1 Department of Medicine I (Cardiology, Angiology, Hemostaseology, Intensive Care), Medical Faculty Mannheim, University of Heidelberg, 68167 Mannheim, Germany; angela.moissl@medma.uni-heidelberg.de (A.P.M.-B.); daniel.duerschmied@umm.de (D.D.); 2 LURIC Study GmbH, Josef-Mörtl-Straße 23, 86482 Aystetten, Germany; graciela.delgado@medma.uni-heidelberg.de (G.E.D.); winfried.maerz@synlab.com (W.M.); 3 Department of Medicine, Medical Faculty Mannheim, University of Heidelberg, 68167 Mannheim, Germany; bernhard.kraemer@umm.de; 4 Federal Institute for Drugs and Medical Services, 53175 Bonn, Germany; ruediger.siekmeier@bfarm.de; 5 Department of Medicine III (Cardiology, Pneumonology, Angiology), Medical Faculty Heidelberg, University of Heidelberg, 69120 Heidelberg, Germany; 6 SYNLAB Academy, SYNLAB Holding Deutschland GmbH, 68161 Mannheim, Germany; 7 SYNLAB MVZ für Humangenetik Mannheim GmbH, 68163 Mannheim, Germany

**Keywords:** Serum Amyloid A (SAA), high-density lipoprotein (HDL), inflammation, cardiovascular disease, cholesterol efflux, oxidative stress, anti-inflammatory therapy, HDL mimetics, nutritional modulation

## Abstract

Serum Amyloid A (SAA) is an acute-phase apolipoprotein that acts as both a sensitive biomarker of systemic inflammation and an active modulator of lipid metabolism and vascular homeostasis. This review summarises current insights into the interaction between SAA and high-density lipoproteins (HDL), with particular emphasis on its role in inflammation-driven cardiovascular disease (CVD). The incorporation of SAA into HDL markedly alters its composition and function. The displacement of apolipoprotein A-I impairs cholesterol efflux capacity, reduces antioxidative activity, and promotes a pro-inflammatory phenotype, transforming protective HDL into a dysfunctional particle. These changes contribute to endothelial dysfunction, foam cell formation, and atherogenesis. Elevated SAA levels are also associated with adverse cardiovascular and metabolic outcomes, including coronary artery disease, type 2 diabetes, and chronic kidney disease. Isoform-specific variations in SAA–HDL interactions are emerging as key modulators of these effects. This review also discusses emerging therapeutic and nutritional strategies to modulate the SAA–HDL axis, including anti-inflammatory therapies, HDL mimetics, and diet-based interventions. Future research should prioritise the standardisation of SAA measurement, characterisation of isoform-specific functions, and translational studies integrating SAA into cardiovascular risk stratification and therapy.

## 1. Introduction

Serum Amyloid A (SAA) belongs to a family of acute-phase apolipoproteins primarily produced by hepatocytes in response to systemic inflammation. It has long been recognised as a sensitive biomarker of systemic inflammatory activity, as thoroughly reviewed by Hosman, Kos, and Lamot [1]. The synthesis of SAA is mainly induced by pro-inflammatory cytokines, including interleukin-6 (IL-6), tumour necrosis factor-α (TNF-α), and interleukin-1β (IL-1β), leading to a significant increase in circulating SAA concentrations—often exceeding a 1000-fold rise during acute-phase responses [2,3,4,5,6].

This pronounced upregulation underscores the role of SAA as both a sensitive biomarker for inflammation and a potential therapeutic target, particularly in cardiovascular disease (CVD) [7].

SAA exists in several isoforms, most notably SAA1 and SAA2, which differ in their expression patterns, structural characteristics, and binding affinities for high-density lipoproteins (HDL). While both are primarily synthesised in the liver, extrahepatic expression—e.g., in macrophages, adipocytes, and endothelial cells—also contributes to local SAA pools, especially under conditions of chronic inflammation. In these settings, SAA may bind not only to HDL but also to other ligands such as glycosaminoglycans and retinol-binding proteins, thereby mediating tissue-specific immunometabolic effects beyond lipoprotein remodelling.

SAA plays a regulatory role in lipid metabolism and vascular homeostasis. Increasing evidence highlights the interaction between SAA and high-density lipoprotein cholesterol (HDL-C) [8]. Incorporation into HDL particles induces structural and functional alterations. Specifically, the displacement of apolipoprotein A-I (ApoA-I) by SAA impairs HDL’s atheroprotective functions, including reverse cholesterol transport (RCT), antioxidative activity, and support of endothelial health [2,5,8,9]. These SAA-induced alterations in HDL function are increasingly recognised as a key mechanistic association between chronic inflammation and atherosclerosis [8,10]. Recent studies, such as those by Speer et al. [10] and Zewinger et al. [11], emphasise the pathological significance of the SAA-HDL interactions, connecting them to foam cell formation, endothelial dysfunction, and the progression of atherosclerotic plaques [7,12].

Consequently, the dual role of SAA as both a biomarker and an active modulator of cardiovascular pathology warrants further investigation. Beyond its traditional classification as an acute-phase reactant, SAA has attracted increasing attention as a dynamic regulator of lipid metabolism and innate immunity [7,8,9]. This review complements recent efforts to characterise inflammation-induced lipoprotein remodelling and expands upon the emerging roles of Serum Amyloid A (SAA) in vascular and metabolic disease.

Under normal conditions, SAA exists only in trace amounts and does not significantly affect the HDL structure or function. However, during acute or chronic inflammation, its rapid and substantial induction profoundly alters the composition and biological activity of circulating lipoproteins. HDL, which is generally regarded as atheroprotective through its roles in reverse cholesterol transport, antioxidative defence, endothelial maintenance, and anti-inflammatory activity, becomes functionally compromised when SAA becomes a predominant apolipoprotein within the particle [9,11,12,13,14]. This shift reflects a broader realisation: inflammation does not merely coexist with dyslipidaemia—it actively reprogrammes lipid metabolism to promote vascular injury [15,16,17].

Importantly, these changes are not confined to patients with obvious inflammatory disease. Even mild, chronic inflammatory activity, as observed in obesity, metabolic syndrome, diabetes, chronic kidney disease, and ageing, can raise SAA concentrations and impair HDL function [16,17,18,19]. This highlights the pivotal role of the SAA–HDL axis as a mechanistic link between immunometabolic dysregulation and cardiovascular disease [8,9,11]. The fact that SAA can serve as both a biomarker and a pathogenic mediator emphasises its significance in preventive cardiology, internal medicine, and translational research.

Therefore, understanding how SAA affects HDL function provides crucial insights into the connection between inflammation and atherosclerosis and identifies potential therapeutic avenues [9,10,11]. This review critically evaluates current knowledge of SAA’s role in inflammation-driven cardiovascular disease, focusing on its interactions with HDL, effects on cholesterol efflux, and implications for therapeutic intervention.

## 2. Relevant Sections

### 2.1. Molecular Mechanisms of the SAA-HDL Interaction

The interaction between Serum Amyloid A (SAA) and high-density lipoprotein (HDL) represents a central mechanism linking inflammation to impaired lipid metabolism and cardiovascular disease. During inflammatory states, hepatic production of SAA increases markedly, and the protein preferentially incorporates into HDL particles, displacing apolipoprotein A-I (ApoA-I) and altering HDL’s structural and functional properties [8,11,18]. This exchange transforms HDL from an atheroprotective lipoprotein into a dysfunctional, pro-inflammatory particle with reduced cholesterol efflux capacity, diminished antioxidative activity, and impaired endothelial-protective effects [12,19,20].

Beyond the well-established displacement of ApoA-I from the HDL surface, SAA induces a broad spectrum of structural and conformational changes that affect HDL’s stability and functionality. SAA-enriched HDL particles tend to become smaller, denser, and compositionally less stable during inflammation. These physical changes reduce the particle’s capacity to interact with cholesterol transporters such as ABCA1 and ABCG1.

#### 2.1.1. Reverse Cholesterol Transport (RCT) and SAA

HDL plays a crucial role in reverse cholesterol transport (RCT), facilitating the removal of excess cholesterol from macrophages and peripheral tissues via ABCA1 and ABCG1 transporters. The displacement of ApoA-I by SAA reduces HDL’s ability to bind these transporters and thereby compromises cholesterol efflux [18,19,21,22].

Experimental data show that SAA-enriched HDL has significantly reduced capacity to promote ABCA1- and ABCG1-mediated efflux, resulting in intracellular cholesterol accumulation, macrophage foam cell formation, and accelerated atherosclerotic plaque progression [12,16,23,24].

These molecular effects of this process are summarised in Table 1.

**Table 1:** Molecular effects of the SAA-HDL interaction on HDL functions.

Function	Effect of Interaction	Clinical Implication	Scientific Sources
Reverse Cholesterol Transport	ApoA-I displacement, reduced cellular cholesterol efflux	Foam cell formation, plaque instability	[15,21,22,25]
Antioxidant capacity	Decreased ROS neutralisation	Endothelial dysfunction, oxidative stress	[7,14,18,23,24]
Anti-inflammatory function	Increased adhesion molecule expression	Vascular inflammation, atherosclerosis	[14,26,27]

#### 2.1.2. Oxidative Stress and Pro-Inflammatory Shift

In addition to impaired cholesterol transport, SAA reduces the antioxidative properties of HDL, leading to increased production of reactive oxygen species (ROS) and oxidative damage to vascular tissues [18,28]. Moreover, dysfunctional HDL upregulates endothelial adhesion molecules such as ICAM-1 and VCAM-1, promoting leukocyte adhesion and transmigration [11,29]. These inflammatory responses contribute to endothelial dysfunction and plaque progression [8,9].

SAA modifies HDL functionality through several interlinked pathways. First, it displaces ApoA-I, which is critical for HDL maturation and cholesterol efflux via the ABCA1 and ABCG1 transporters. This displacement diminishes HDL’s ability to remove cholesterol from macrophages, favouring foam cell formation and plaque progression. Second, SAA-bound HDL loses its ability to neutralise reactive oxygen species (ROS), thereby increasing oxidative stress and contributing to endothelial dysfunction. Third, SAA promotes a pro-inflammatory HDL phenotype by upregulating adhesion molecules, such as ICAM-1 and VCAM-1, on endothelial cells, thereby facilitating leukocyte recruitment and vascular inflammation. SAA affects HDL particles mechanically through several interlinked pathways [10,28]. First, it displaces ApoA-I, which is critical for HDL maturation and cholesterol efflux via the ABCA1 and ABCG1 transporters [20]. This displacement diminishes HDL’s ability to remove cholesterol from macrophages, favouring foam cell formation and plaque progression [12,20,30,31]. Second, SAA-bound HDL loses its ability to neutralise reactive oxygen species (ROS), thereby increasing oxidative stress and contributing to endothelial dysfunction [2,16,20]. Third, SAA promotes a pro-inflammatory HDL phenotype by upregulating adhesion molecules such as ICAM-1 and VCAM-1 on endothelial cells, facilitating leukocyte recruitment and vascular inflammation [9,11,32].

Beyond the well-established displacement of ApoA-I from the HDL surface, SAA induces a broad spectrum of structural and conformational changes that compromise HDL’s stability and functionality [33,34,35]. During inflammation, SAA-enriched HDL particles typically become denser, smaller, and compositionally less stable [11,18,20]. These physical changes reduce the particle’s capacity to interact with cholesterol transporters such as ABCA1 and ABCG1 [16,26,31]. While ApoA-I is optimally structured for high-affinity binding to these transporters, SAA lacks the amphipathic helix architecture required for efficient cholesterol mobilisation [6,7,18]. Therefore, macrophages retain cholesterol and transition more readily into foam cells, contributing to plaque growth and instability [12,21,23].

SAA incorporation also has significant implications for HDL-associated enzymes [14,20]. Paraoxonase-1 (PON1), a key antioxidative enzyme carried primarily on HDL, becomes displaced or functionally inhibited in the presence of high SAA concentrations [34,36,37].

Reduced PON1 activity impairs HDL’s ability to neutralise oxidised lipids and prevent oxidative modification of LDL, which in turn accelerates endothelial injury and plaque formation [17]. This loss of antioxidative function represents a major driver of oxidative stress in the vascular wall and facilitates a pro-atherogenic environment.

Similarly, lecithin–cholesterol acyltransferase (LCAT), which is responsible for HDL maturation, is less effective when SAA disrupts HDL’s surface lipid environment. This impairs HDL particle maturation and limits its role in reverse cholesterol transport, further contributing to lipid accumulation in vascular lesions. This hampers particle maturation and limits HDL’s role in reverse cholesterol transport, exacerbating lipid accumulation in vascular lesions. Consequently, there is an increased proportion of immature, poorly functional HDL particles in the circulation [38].

Another relevant aspect is SAA’s ability to recruit and activate pattern-recognition receptors. SAA can engage Toll-like receptors (TLR2 and TLR4), formyl peptide receptor-like 1 (FPRL1), and other innate immune sensors, amplifying vascular inflammation. Through these receptors, SAA stimulates NF-κB signalling, increases cytokine production, and enhances endothelial adhesion molecule expression. These pathways promote a sustained inflammatory response and link innate immune signalling directly to vascular damage and plaque vulnerability.

HDL enriched in SAA appears to lose its buffering capacity against these inflammatory cascades, further intensifying the inflammatory milieu within the vascular wall [8,39]. SAA additionally affects lipid rafts and membrane microdomains in target cells.

By altering the distribution of cholesterol and sphingolipids in the plasma membrane, it may enhance the sensitivity of endothelial and immune cells to inflammatory stimuli. This can potentiate leukocyte recruitment, endothelial permeability, and smooth muscle cell activation—all of which are central to the development of vulnerable atherosclerotic plaques [17,22]. Beyond these direct effects, SAA-HDL interactions also appear to influence intracellular signalling pathways that shape vascular and immune responses [16,31]. Experimental studies indicate that SAA-enriched HDL can alter endothelial nitric oxide synthase (eNOS) activity, reducing nitric oxide bioavailability and impairing vasodilation. Reduced nitric oxide contributes not only to endothelial dysfunction but also to enhanced oxidative stress, creating a self-perpetuating cycle of vascular injury [40].

Furthermore, SAA-bound HDL has been shown to modulate macrophage phenotype and lipid handling [22,41]. By impairing cholesterol efflux and promoting intracellular lipid accumulation, SAA fosters a transition of macrophages toward a more inflammatory, foam cell–prone state. This phenotypic shift reinforces local inflammation within atherosclerotic lesions and contributes to plaque instability. Another important aspect is the impact of SAA on HDL proteome and lipidome composition [14,18,19]. SAA-rich HDL exhibits an altered complement of proteins involved in antioxidative defence, acute-phase signalling, and lipid metabolism. These changes impair HDL’s functional plasticity and may undermine its adaptive responses under inflammatory stress.

Taken together, these multilayered alterations underscore the complex and dynamic nature of HDL dysfunction in the presence of SAA, extending far beyond simple apolipoprotein displacement [11].

**Figure 1:**
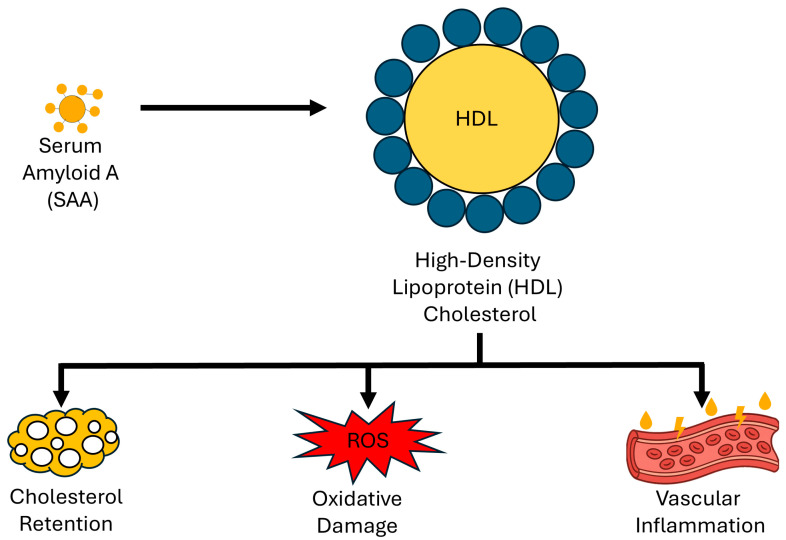
Simplified mechanistic diagram illustrating SAA-HDL interaction and downstream effects.

These interconnected effects are summarised in Table 2, which outlines the principal mechanisms by which SAA transforms HDL into a dysfunctional and potentially atherogenic lipoprotein. To complement this overview, Figure 1 provides a simplified schematic illustration of the significant molecular consequences of SAA-HDL interaction.

**Table 2:** Mechanistic insights into SAA-HDL interaction and associated pathophysiological outcomes.

Mechanisms	Effect of SAA on HDL	Outcome	References
Reverse cholesterol transport	ApoA-1 displacement	Foam cell formation, plaque instability	[15,17,29,31,32]
Antioxidant function	Reduced ROS neutralisation	Oxidative stress, endothelial dysfunction	[2,6,7,14]
Anti-inflammatory function	Increased adhesion molecule expression	Leucocyte recruitment, vascular inflammation	[7,39,42,43,44]

In light of these findings, future therapeutic strategies may focus on reducing systemic SAA concentrations and stabilising HDL structure during inflammation. Nutritional interventions—such as omega-3 fatty acids or polyphenol-rich diets—may offer supportive benefits by enhancing HDL resilience and suppressing SAA synthesis [23,24]. A comprehensive therapeutic model should ideally combine pharmacological, biological, and nutritional elements to counteract the multifactorial impact of SAA on HDL functionality and cardiovascular risk.

### 2.2. Clinical Significance of SAA-Mediated HDL Dysfunction

Elevated levels of SAA have been consistently linked to increased cardiovascular and metabolic risk [45]. In the context of cardiovascular disease, numerous studies have demonstrated that high circulating SAA levels are independently associated with a higher risk of major adverse cardiovascular events (MACE), including myocardial infarction, stroke, and cardiovascular mortality [9,11,19].

This makes SAA a valuable biomarker for inflammation and cardiovascular risk stratification, particularly in patients with underlying chronic disease or acute coronary syndromes [7].

Beyond its role in cardiovascular pathology, SAA has also emerged as a key biomarker in metabolic diseases such as type 2 diabetes and chronic kidney disease [2,32,41,46].

These conditions are often characterised by persistent low-grade inflammation and dysfunctional lipoprotein metabolism. Elevated SAA levels in these patients correlate with impaired HDL function, reduced cholesterol efflux, and increased oxidative stress, contributing to endothelial damage and micro- and macrovascular complications [31,33].

Chang et al. highlighted the prognostic relevance of SAA in predicting vascular complications and its potential utility in metabolic risk profiling [47]. Owing to its dual role as a biomarker and mediator of vascular pathology, SAA represents a promising target for risk stratification and personalised intervention in cardiovascular and metabolic diseases. Its incorporation into established risk assessment models—particularly for patients with inflammatory or metabolic disorders—offers the potential to improve early identification of high-risk individuals, guide tailored therapeutic strategies, and enhance clinical outcomes [36]. The spectrum of clinical conditions associated with SAA-HDL dysfunction is summarised in Table 3.

The clinical implications of these mechanistic changes extend beyond classical cardiovascular endpoints. Elevated SAA reflects a state of heightened inflammatory activity that simultaneously affects multiple organ systems [7,8,35,46].

In patients with chronic inflammatory diseases, the combination of persistent SAA elevation and dysfunctional HDL creates a milieu in which endothelial repair mechanisms are insufficient to counteract cumulative vascular injury. This may help explain why even patients with optimal LDL-cholesterol control continue to experience a residual inflammatory risk, leading to recurrent cardiovascular events despite standard therapy [33,48,49,50].

Beyond chronic disease associations, SAA’s clinical relevance also extends to dynamic inflammatory states and short-term cardiovascular risk.

Furthermore, SAA levels respond rapidly to fluctuations in inflammatory activity, making it a sensitive and dynamic marker for short-term risk assessment. Patients undergoing acute infections, major surgery, or metabolic decompensation often exhibit transient surges in SAA, which may impair HDL function and contribute to short-term increases in thrombotic and vascular complications [7,35]. This reactivity distinguishes SAA from more static markers such as LDL-cholesterol and suggests potential value in longitudinal monitoring [48].

There is also growing recognition that SAA might contribute to microvascular dysfunction in metabolic and renal diseases [51]. Microvascular complications in diabetes and chronic kidney disease, such as retinopathy or endothelial rarefaction, may be partly due to chronic exposure to dysfunctional HDL enriched with SAA [45,52,53]. This is consistent with clinical observations showing that patients with persistent inflammation, even at low grade, have disproportionate impairment of HDL-mediated cholesterol efflux and antioxidative capacity [20,33,44].

In addition to these associations, the relationship between SAA and cardiometabolic risk appears to be modulated by demographic and lifestyle factors.

Ageing, for example, is associated with a gradual increase in basal inflammatory tone (“inflammaging”), which may render older individuals more susceptible to SAA-induced HDL dysfunction [17,54]. Similarly, obesity and visceral adiposity are characterised by chronic low-grade inflammation, leading to persistent SAA elevations even in the absence of overt disease [18,52]. This chronic inflammatory burden may amplify HDL dysfunction and accelerate vascular injury, particularly in individuals with metabolic syndrome. Emerging evidence also suggests that SAA may serve as a molecular link between psychosocial stress, neuroendocrine activation, and cardiovascular risk [16,31]. Stress-induced cytokine release can upregulate hepatic SAA production, contributing to acute alterations in HDL quality [7,35,47]. These observations highlight the multifaceted nature of SAA as both a biomarker and functional effector that integrates metabolic, endocrine, and immunological pathways [1,52,53,54,55].

Understanding these interactions may help identify patient subgroups who would benefit most from targeted therapeutic strategies aimed at restoring HDL functionality [19,56]. In this context, SAA may serve as a valuable adjunct biomarker to conventional lipid parameters, particularly for identifying residual cardiovascular inflammatory risk despite optimal LDL-cholesterol control.

**Table 3:** Clinical conditions associated with SAA-HDL interactions.

Condition	Mechanism of Involvement	Clinical Outcome	Scientific Sources
Coronary artery disease	Plaque instability, oxidative stress	Increased infarction risk	[1,25,50,57]
Rheumatoid arthritis	Accelerated atherosclerosis	Higher cardiovascular risk	[1,42,58,59,60]
Type 2 diabetes mellitus	Impaired cholesterol transport	Microangiopathy, insulin resistance	[44,45,48]
Chronic Kidney Disease	Reduced HDL function	Increased cardiovascular mortality	[20,59,61,62,63]

### 2.3. Therapeutic Strategies Targeting SAA-HDL Interactions

Therapeutic strategies aimed at modulating the deleterious interaction between Serum Amyloid A (SAA) and high-density lipoprotein (HDL) are of increasing clinical interest, particularly in the prevention of cardiovascular and metabolic diseases [64]. Because SAA binding compromises HDL’s core protective functions—including reverse cholesterol transport (RCT), antioxidative capacity, and anti-inflammatory activity—targeting this interaction represents a rational and multifaceted therapeutic approach [9,36,39,52,65]. Among the best-established pharmacological interventions to prevent cardiovascular events, statins are well recognised for their lipid-lowering and anti-inflammatory properties. Statin therapy can moderately reduce circulating SAA levels, which correlates with partial restoration of HDL function. However, statins appear insufficient to fully reverse SAA-associated HDL dysfunction, particularly in patients with persistent systemic inflammation [20]. Consequently, their role in this context is likely supportive rather than curative.

Biological agents that directly inhibit inflammatory cytokines upstream of SAA synthesis—most notably interleukin-6 (IL-6) and tumour necrosis factor-α (TNF-α)—have demonstrated more pronounced effects [52]. By reducing hepatic SAA production, these agents may indirectly preserve HDL structure and functionality. Studies have shown that IL-6 blockade, for example, leads to substantial reductions in SAA levels and attenuation of vascular inflammation, thereby potentially improving cholesterol efflux capacity and endothelial integrity [8,14]. Such biologic therapies may be particularly beneficial in chronic inflammatory conditions, including rheumatoid arthritis, where SAA and HDL dysfunction are elevated [6]. Moreover, recent observations suggest that SAA levels may rise markedly during cytokine release syndromes, such as those seen in CAR-T cell therapies. In these acute inflammatory settings, SAA may function not only as a dynamic marker of systemic inflammation but also as an active contributor to acute vascular complications. This underscores the potential relevance of targeting SAA and its upstream mediators even in short-term, high-intensity inflammatory states [1,66,67]. There is also compelling clinical evidence that targeting subclinical inflammation, along with downstream inflammatory effectors, reduces cardiovascular events. The Canakinumab Anti-Inflammatory Thrombosis Outcomes Study (CANTOS) demonstrated that interleukin-1β (IL-1β) inhibition significantly lowers cardiovascular risk in patients with residual inflammatory burden [40,56]. Although SAA was not directly assessed as a trial endpoint, IL-1 blockade is expected to reduce hepatic SAA synthesis and may thereby indirectly improve HDL functionality and vascular protection. The extent to which reductions in SAA contributed to the observed clinical benefit remains unclear. In parallel, ongoing trials are evaluating IL-6 inhibition with agents such as ziltivekimab (NCT05021835) and clazakizumab (NCT05485961) for cardiovascular risk reduction in patients with chronic kidney disease, a population characterised by prevalent subclinical inflammation. In addition to cytokine-targeted therapies, emerging strategies include HDL mimetics—engineered peptides, liposomes, or nanoparticle-based constructs designed to replicate the functional properties of native HDL. Preclinical studies have demonstrated that HDL-mimetic nanoparticles can restore RCT in the presence of elevated SAA levels, enhance macrophage cholesterol efflux, and reduce plaque size and instability [60]. Nevertheless, clinical outcomes with HDL mimetics and HDL-raising agents, such as cholesteryl ester transfer protein (CETP) inhibitors, have thus far been disappointing [5,68,69].

Beyond cytokine inhibition and HDL mimetics, a key therapeutic concept is the preservation or restoration of HDL functionality rather than simply increasing HDL cholesterol (HDL-C) levels. Potential approaches include stabilising ApoA-I to prevent its displacement by SAA, as well as developing peptide inhibitors or monoclonal antibodies that disrupt the SAA–HDL interface. Interventions targeting nuclear factor κB (NFκB) or IL-6 signalling may further reduce hepatic SAA synthesis and attenuate acute-phase remodelling of HDL [7,8,17]. Collectively, these findings indicate that although multiple strategies can mechanistically improve HDL function or reduce SAA levels, consistent translation into clinical benefit remains limited [1]. This highlights the need for more personalised, multimodal therapeutic approaches that address both inflammatory drivers and HDL dysfunction. Lifestyle-based interventions remain central in this context. Regular physical activity, weight reduction, and improved glycaemic control have been shown to enhance HDL functionality even in the presence of systemic inflammation [70,71,72,73]. Exercise-induced myokines may further suppress cytokine-driven SAA production and partially restore HDL function [24,44,53].

### 2.4. Nutritional Modulation of SAA and HDL Function

Diet plays a central role in maintaining lipid homeostasis and regulating inflammation [60,74,75]. Recent evidence highlights a critical role of nutrition in modulating SAA expression and HDL functionality. Several studies have demonstrated that specific dietary patterns and bioactive nutrients can influence the SAA–HDL axis in clinically meaningful ways [19]. Among these, the Mediterranean diet has received special attention.

Characterised by a high intake of fruits, vegetables, whole grains, olive oil, nuts, and fatty fish, this dietary pattern reduces systemic inflammation and improves HDL quality [76,77]. Adherence to the Mediterranean diet has been associated with lower circulating SAA concentrations and enhanced HDL-mediated cholesterol efflux [53]. These effects are thought to be due to the high content of polyunsaturated fatty acids (PUFAs), especially omega-3 fatty acids such as eicosapentaenoic acid (EPA) and docosahexaenoic acid (DHA), which modulate pro-inflammatory cytokine signalling and suppress hepatic SAA synthesis [24,77].

**Figure 2:**
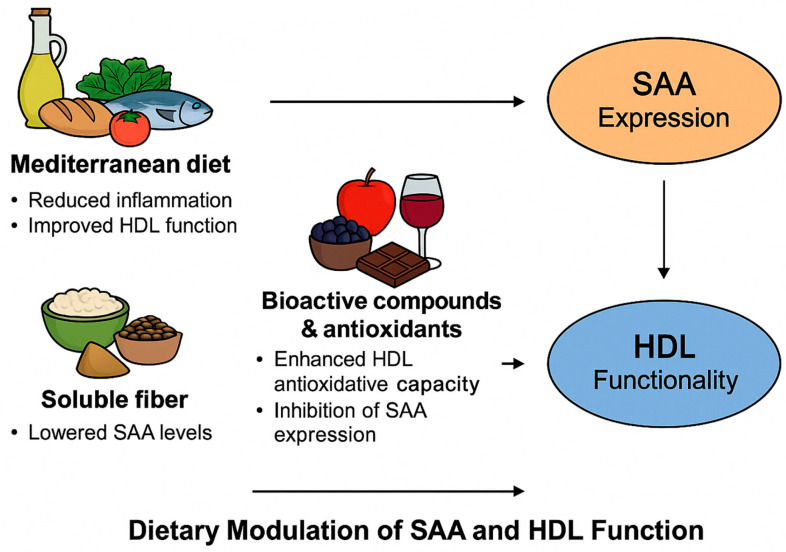
Dietary influences on the SAA-HDL axis. Key nutritional components—including the Mediterranean diet, bioactive compounds, and soluble fibre—modulate SAA expression and HDL functionality through anti-inflammatory, antioxidative, and metabolic pathways.

In addition to the fatty acid composition, Dietary polyphenols appear to exert protective effects on HDL structure and function [74]. Polyphenol-rich foods, such as berries, green tea, dark chocolate, and red wine, preserve HDL’s antioxidative capacity and may attenuate SAA expression [75]. Flavonoids such as quercetin and resveratrol have been shown to enhance the expression of key cholesterol transporters, including ABCA1, and to protect HDL from oxidative modification, thereby maintaining endothelium-protective functions [60]. Soluble dietary fibre, particularly from oats, legumes, and flaxseeds, supports HDL functionality through indirect anti-inflammatory mechanisms [14,60,74]. Dietary fibre modulates gut microbiota composition, promoting the production of short-chain fatty acids (SCFAs), such as butyrate, which exert systemic anti-inflammatory effects and may reduce circulating SAA levels [60,74,78,79,80]. These findings underscore the potential of fibre-rich diets in mitigating inflammation-associated HDL dysfunction (Figure 2).

Collectively, these observations suggest that targeted nutritional strategies may serve as effective adjuncts to pharmacological therapies in the management of SAA-mediated HDL dysfunction [74]. However, despite compelling mechanistic insights, most evidence linking dietary patterns to SAA modulation derives from observational studies or small clinical trials. Heterogeneity in study design, endpoints, and biomarker selection limits direct comparability, and confounding lifestyle factors may influence outcomes. Therefore, well-designed, controlled clinical trials are required to establish causality and define optimal dietary interventions to reduce inflammation and restore HDL function in at-risk populations. In clinical practice, nutritional approaches may be particularly valuable for patients who do not tolerate anti-inflammatory pharmacotherapy or in whom residual inflammatory risk persists despite optimal medical treatment.

### 2.5. Interplay Between SAA, HDL, and the Innate Immune System

The interaction between SAA, HDL, and the innate immune system represents an essential and increasingly recognised component of inflammation-driven cardiovascular pathology. While the molecular effects of SAA on HDL structure and function are well established, their broader implications for innate immune signalling, vascular inflammation, and metabolic dysregulation are increasingly evident. These interactions highlight the multifaceted role of SAA as both a biomarker and an active modulator of immune–metabolic processes, contributing to HDL dysfunction and atherosclerotic progression in a wide range of clinical contexts [7,39,61]. This section focuses specifically on the immunological and systemic consequences of SAA-HDL interactions, in contrast to the primarily mechanistic focus in Section 2.1.

A central aspect of this interplay is SAA’s ability to engage pattern-recognition receptors (PRRs), including Toll-like receptors (TLR2, TLR4) and formyl peptide receptor-like 1 (FPRL1) [81]. These receptors serve as key sensors of inflammatory danger signals and pathogen-associated molecular patterns. By activating these receptors, SAA can directly initiate intracellular signalling cascades such as NF-κB and MAP kinase pathways, promoting the expression of cytokines, chemokines, and adhesion molecules [39,82]. Notably, SAA-enriched HDL appears unable to buffer or neutralise these inflammatory signals, thereby amplifying the inflammatory response and contributing to endothelial dysfunction [10,83].

In macrophages, SAA-driven signalling promotes classical (M1-like) activation, characterised by increased production of pro-inflammatory mediators and enhanced uptake of modified lipids. This pro-inflammatory macrophage phenotype is particularly relevant in the context of atherosclerosis, where SAA promotes pro-inflammatory macrophage activation and foam cell accumulation, reinforcing innate immune activation and tissue inflammation [22,25].

The sustained presence of SAA in the inflammatory microenvironment promotes macrophage retention and accelerates plaque progression, contributing to necrotic core expansion and plaque instability [11].

Within endothelial cells, SAA mediates the activation of TLRs, including ICAM-1, VCAM-1, and E-selectin. These molecules facilitate leukocyte adhesion and transmigration, intensifying the influx of monocytes into the vessel wall [16,30,31,84].

SAA-induced endothelial dysfunction, marked by reduced nitric oxide bioavailability, contributes to a pro-inflammatory and pro-thrombotic vascular state [42,85,86]. Collectively, these processes create a feed-forward loop in which chronic inflammation results in persistent structural and functional impairment of the vascular endothelium [30,31].

Neutrophils also appear to be important targets of SAA activity. SAA can stimulate neutrophil chemotaxis, degranulation, and respiratory burst activity. In addition, SAA has been implicated in the formation of neutrophil extracellular traps (NETs), which contribute to thrombosis, endothelial damage, and impaired lipoprotein function [6].

NET-associated proteases can degrade HDL-associated proteins, further compromising their immunomodulatory capacity and exacerbating vascular injury [17,87].

The interaction between SAA and HDL also affects the complement system, another essential component of innate immunity. SAA-enriched HDL has been shown to carry higher levels of complement components and acute-phase proteins, suggesting an altered functional profile that may shift HDL from an anti-inflammatory molecule toward a pro-inflammatory carrier [88]. These compositional changes may enable HDL to participate in complement activation, potentially contributing to vascular inflammation and endothelial injury.

Beyond its direct effects on immune cells, SAA plays an important role in modulating lipid rafts, highly ordered membrane microdomains that serve as platforms for receptor clustering and signal transduction. By redistributing cholesterol and sphingolipids within the membrane, SAA can enhance the sensitivity of immune and endothelial cells to inflammatory stimuli [7]. This facilitates the assembly of signalling complexes, amplifying downstream inflammatory responses and contributing to vascular dysfunction.

Interactions between SAA, HDL, and adipose tissue-derived inflammatory mediators further emphasise the systemic relevance of this axis [70].

In obesity and metabolic syndrome, hypertrophic adipocytes secrete pro-inflammatory cytokines that stimulate hepatic SAA production [89]. Elevated SAA, in turn, exacerbates adipose tissue inflammation by promoting macrophage infiltration and impairing lipid handling in adipocytes, contributing to insulin resistance and metabolic dysregulation [1,44,70]. These observations illustrate a reciprocal relationship between metabolic inflammation and SAA-driven HDL dysfunction, emphasising the significance of this axis in the progression of cardiometabolic disease [70,90].

A growing area of interest concerns the gut–liver axis and its influence on SAA expression and HDL function. Alterations in gut microbiota composition, often observed in obesity, diabetes, and chronic inflammatory diseases, can modulate systemic cytokine levels and hepatic acute-phase protein synthesis [66,91]. Increased intestinal permeability and the translocation of microbial products, such as lipopolysaccharides (LPS), may further stimulate hepatic SAA production via TLR-mediated signalling. In this setting, SAA-driven HDL dysfunction contributes to impaired cholesterol metabolism, enhanced vascular inflammation, and accelerated atherogenesis [11,44,92].

SAA-driven remodelling accelerates HDL turnover, potentially limiting its immunoregulatory availability during chronic inflammation [93]. During acute and chronic inflammation, accelerated HDL catabolism and reduced HDL lifespan have been reported, potentially mediated by SAA-driven compositional changes that reduce particle stability [94,95,96]. This may further limit the availability of functional HDL capable of performing cholesterol efflux and antioxidative functions, exacerbating the negative impact of inflammation on lipid homeostasis.

Taken together, these findings underscore the complex interplay between SAA, HDL, and the innate immune system as a critical driver of inflammation-associated cardiovascular and metabolic disease. SAA does not merely mark inflammatory activity but actively participates in shaping immune cell function, endothelial behaviour, and lipoprotein composition. Its effects are amplified under conditions of chronic low-grade inflammation, linking metabolic dysfunction, immune activation, and vascular pathology [9,65]. A deeper understanding of these interactions may reveal novel therapeutic opportunities to break the self-sustaining cycle of inflammation, lipoprotein dysfunction, and cardiovascular risk. Clarifying which immune responses are causally driven by SAA-enriched HDL versus those that are secondary to inflammation remains an important goal for future research.

### 2.6. Future Directions and Research Gaps

Despite significant advances in understanding the role of SAA in HDL dysfunction and cardiovascular disease, several essential questions remain unanswered. First, the functional differences between SAA isoforms, particularly SAA1 and SAA2, are not yet fully defined. While general SAA—predominantly SAA1 and SAA2—can replace ApoA-I on HDL during inflammation and is exchangeable among lipoproteins, this raises questions about the biological relevance of isoform-specific effects that are currently underexplored in clinical settings. Further studies are needed to determine whether these binding differences translate into distinct functional or prognostic outcomes in vivo [68,97].

Second, the long-term prognostic relevance of SAA levels in diverse and multi-morbid patient populations requires validation in prospective, longitudinal studies [55].

Third, the absence of standardised and clinically validated assays for SAA measurement hampers its integration into routine diagnostics. Moreover, several analytical and methodological challenges limit the clinical application of SAA testing. Most commercially available assays rely on polyclonal antibodies that lack isoform specificity, making it difficult to distinguish between SAA1 and SAA2 or between intact proteins and degradation products. Furthermore, the absence of a universal reference standard complicates interlaboratory comparability. Enzyme-linked immunosorbent assays (ELISA) offer high sensitivity but are time-consuming, whereas immunonephelometric and immunoturbidimetric assays, while automated and rapid, generally have lower sensitivity (limit of detection typically > 3 mg/L). A recently introduced chemiluminescence immunoassay (CLIA) may offer improved analytical performance, but broader clinical validation is still pending [98].

In addition, many mechanistic studies use recombinant, lipid-free SAA in vitro. However, this form does not fully recapitulate the physiological behaviour of SAA, which circulates predominantly in HDL-bound form in vivo. This discrepancy may limit the translational relevance of findings related to cholesterol efflux, oxidative stress, or receptor activation. Future studies should aim to employ more physiologically relevant models that better reflect SAA’s interaction with HDL in the native circulatory context.

Finally, the clinical efficacy of interventions specifically targeting the SAA–HDL axis, particularly in chronically inflamed, high-risk populations, remains to be determined [17,64].

To date, no interventional trial has directly targeted this axis, and most data derive from observational or preclinical models. This lack of translational evidence limits clinical implementation. A more nuanced understanding of these aspects will be essential to translate molecular insights into targeted, evidence-based clinical strategies. A deeper understanding of how SAA affects HDL function in different pathological contexts will be crucial for overcoming current limitations in cardiovascular risk prediction. Future research should also aim to characterise the temporal dynamics of SAA elevation across various diseases and to determine how acute spikes versus chronic elevations differentially affect HDL composition and cardiovascular outcomes [10,19]. Another key area concerns the interplay between genetic factors and inflammatory drivers of SAA expression. Polymorphisms in SAA1 and SAA2 may influence both expression levels and HDL-binding properties, thereby modifying individual susceptibility to inflammation-induced HDL dysfunction [14]. However, the clinical implications of these polymorphisms remain largely speculative, and their interactions with environmental or metabolic stressors are poorly understood.

Moreover, integrating SAA measurements into multi-marker panels combining lipidomics, proteomics, and inflammatory profiling may substantially enhance risk stratification. Such panels could help identify patients in whom HDL dysfunction is primarily driven by inflammation, enabling personalised treatment strategies [97].

Closing this translational gap will require coordinated efforts integrating molecular research, biomarker standardisation, and clinical trial validation.

## 3. Conclusions

This review underscores the central role of Serum Amyloid A (SAA) as both a marker and mediator of inflammation-induced cardiovascular risk. By binding to HDL and altering its structural organisation, SAA impairs key atheroprotective functions, including reverse cholesterol transport, antioxidative defence, and modulation of endothelial activation. These effects extend beyond acute inflammatory states and are increasingly recognised in chronic conditions such as diabetes, obesity, chronic kidney disease, autoimmune disorders, and ageing. The cumulative burden of low-grade inflammation may therefore contribute to persistent HDL dysfunction and residual cardiovascular risk, even in individuals receiving optimal lipid-lowering therapy.

The emerging mechanistic understanding of the SAA–HDL axis provides a compelling rationale for therapeutic interventions that not only lower LDL cholesterol but also preserve or restore HDL quality. Anti-inflammatory agents, HDL mimetics, and lifestyle or nutritional interventions represent promising but still evolving strategies. However, substantial gaps remain, particularly in translating mechanistic insights into clinical application. These include the need for standardised SAA assays, better characterisation of the distinct biological roles of SAA isoforms—particularly SAA1 vs. SAA2—in different inflammatory and metabolic contexts, and longitudinal studies to clarify how fluctuations in SAA influence cardiovascular outcomes over time. In particular, interventional studies directly targeting the SAA–HDL interface is lacking, and it remains unclear whether reducing SAA levels alone suffices to restore HDL function or improve outcomes.

Future research that integrates molecular, metabolic, and clinical perspectives will be essential for translating these insights into personalised prevention and treatment strategies. Ultimately, advancing our understanding of SAA-mediated HDL dysfunction may help refine cardiovascular risk assessment, improve therapeutic decision-making, and ultimately break the cycle of chronic inflammation, lipoprotein dysfunction, and residual cardiovascular risk in vulnerable populations.

## Data Availability

No new data were created or analysed in this study. Data sharing is not applicable to this article.

## Abbreviations

The following abbreviations are used in this manuscript:

ABCA1	ATP-binding cassette transporter A1
ABCG1	ATP-binding cassette transporter G1
ACS	Acute Coronary Syndrome
AHA	American Heart Association
ApoA-I	Apolipoprotein A-I
AUC	Area Under the Curve
BMI	Body Mass Index
CAD	Coronary Artery Disease
CKD	Chronic Kidney Disease
CKD-EPI-GFR	Chronic Kidney Disease Epidemiology Collaboration Estimated Glomerular Filtration Rate
CRP	C-Reactive Protein
CV	Cardiovascular
CVD	Cardiovascular Disease
DHA	Docosahexaenoic Acid
ELISA	Enzyme-Linked Immunosorbent Assay
EPA	Eicosapentaenoic Acid
ESC/ESH	European Society of Cardiology/European Society of Hypertension
HDL-C	High-Density Lipoprotein Cholesterol
hsCRP	High-Sensitivity C-Reactive Protein
ICAM-1	Intercellular Adhesion Molecule 1
IL-1β	Interleukin-1 beta
IL-6	Interleukin-6
LDL	Low-Density Lipoprotein
LURIC	Ludwigshafen Risk and Cardiovascular Health Study
MACE	Major Adverse Cardiovascular Events
MI	Myocardial Infarction
NT-proBNP	N-terminal pro-B-type Natriuretic Peptide
PAD	Peripheral Artery Disease
PRISMA	Preferred Reporting Items for Systematic Reviews and Meta-Analyses
PUFA	Polyunsaturated Fatty Acids
RCT	Reverse Cholesterol Transport
ROC	Receiver Operating Characteristic
ROS	Reactive Oxygen Species
SAA	Serum Amyloid A
SCFA	Short-Chain Fatty Acids
SD	Standard Deviation
SPSS	Statistical Package for the Social Sciences
TNF-α	Tumour Necrosis Factor-alpha
VCAM-1	Vascular Cell Adhesion Molecule 1
